# Supplementary material for: Using machine learning methods to predict all-cause somatic hospitalizations in adults: A systematic review
Source: PLoS One. 2024 Aug 23;19(8):e0309175. doi: 10.1371/journal.pone.0309175 (PMC11343463; doi:10.1371/journal.pone.0309175)
Supplement: S1 File — (DOCX) [file pone.0309175.s001.docx]

**Supplementary 1 (S1)**

**For the publication “Using machine learning methods to predict all-cause somatic hospitalizations in adults: A systematic review.”**

Supplementary contents

[*1. PRISMA 2020 Checklists 2*](#_Toc167373803)

[*2. Inclusions and exclusions criteria 6*](#_Toc167373804)

[*3. Literature search syntax 8*](#_Toc167373805)

[*4. Data sources and their frequency of use in the included studies 17*](#_Toc167373806)

[*5. Benchmarking with risk indexes 20*](#_Toc167373807)

[*6. On the generalizability of ML models 21*](#_Toc167373808)

[*7. A suggestion for a reporting checklist for ML models in structured datasets 22*](#_Toc167373809)

[*References 26*](#_Toc167373810)

# 1. PRISMA 2020 Checklists

| **Section and Topic** | **Item #** | **Checklist item** | **Location where item**  **is reported** |
| --- | --- | --- | --- |
| **TITLE** | | |  |
| Title | 1 | Identify the report as a systematic review. | Title, page 1 |
| **ABSTRACT** | | |  |
| Abstract | 2 | See the PRISMA 2020 for Abstracts checklist. | Abstract, page 1  (PRISMA-Abstract follows as an attachment) |
| **INTRODUCTION** | | |  |
| Rationale | 3 | Describe the rationale for the review in the context of existing knowledge. | Intro., page 2 |
| Objectives | 4 | Provide an explicit statement of the objective(s) or question(s) the review addresses. | Intro., page 2 |
| **METHODS** | | |  |
| Eligibility criteria | 5 | Specify the inclusion and exclusion criteria for the review and how studies were grouped for the syntheses. | Methods, page 3. Supp.1.S2 |
| Information sources | 6 | Specify all databases, registers, websites, organisations, reference lists and other sources searched or consulted to identify studies. Specify the date when each source was last searched or consulted. | Methods, page 3 |
| Search strategy | 7 | Present the full search strategies for all databases, registers and websites, including any filters and limits used. | Supp.1.S3 |
| Selection process | 8 | Specify the methods used to decide whether a study met the inclusion criteria of the review, including how many reviewers screened each record and each report retrieved, whether they worked independently, and if applicable, details of automation tools used in the process. | Methods, page 3-4 |
| Data collection process | 9 | Specify the methods used to collect data from reports, including how many reviewers collected data from each report, whether they worked independently, any processes for obtaining or confirming data from study investigators, and if applicable, details of automation tools used in the process. | Methods, page 3-4  Supp.1.S2 |
| Data items | 10a | List and define all outcomes for which data were sought. Specify whether all results that were compatible with each outcome domain in each study were sought (e.g. for all measures, time points, analyses), and if not, the methods used to decide which results to collect. | Methods, page 3 |
|  | 10b | List and define all other variables for which data were sought (e.g. participant and intervention characteristics, funding sources). Describe any assumptions made about any missing or unclear information. | Supp.2 (Sheet:  CHARMS) |
| Study risk of bias assessment | 11 | Specify the methods used to assess risk of bias in the included studies, including details of the tool(s) used, how many reviewers assessed each study and whether they worked independently, and if applicable, details of automation tools used in the process. | Methods, page 4  Supp.2 (Sheet:  PROBAST) |
| Effect measures | 12 | Specify for each outcome the effect measure(s) (e.g. risk ratio, mean difference) used in the synthesis or presentation of results. | Supp.2 (Sheet:  CHARMS) |
| Synthesis methods | 13a | Describe the processes used to decide which studies were eligible for each synthesis (e.g. tabulating the study intervention characteristics and comparing against the planned groups for each synthesis (item #5)). | Not relevant (The review is descriptive) |
|  | 13b | Describe any methods required to prepare the data for presentation or synthesis, such as handling of missing summary statistics, or data conversions. | Methods, page 4 |
|  | 13c | Describe any methods used to tabulate or visually display results of individual studies and syntheses. | Methods, page 4 |
|  | 13d | Describe any methods used to synthesize results and provide a rationale for the choice(s). If meta-analysis was performed, describe the model(s), method(s) to identify the presence and extent of statistical heterogeneity, and software package(s) used. | No meta-analysis |
|  | 13e | Describe any methods used to explore possible causes of heterogeneity among study results (e.g. subgroup analysis, meta-regression). | Discussion, page 11-12 |
|  | 13f | Describe any sensitivity analyses conducted to assess robustness of the synthesized results. | Not relevant |
| Reporting bias | 14 | Describe any methods used to assess risk of bias due to missing results in a synthesis (arising from reporting biases). | Not relevant |

| **Section and Topic** | **Item #** | **Checklist item** | **Location where item**  **is reported** |
| --- | --- | --- | --- |
| Certainty assessment | 15 | Describe any methods used to assess certainty (or confidence) in the body of evidence for an outcome. | Supp.2 Sheet (CHARMS, PROBAST, TRIPOD) |
| **RESULTS** | | |  |
| Study selection | 16a | Describe the results of the search and selection process, from the number of records identified in the search to the number of studies included in the review, ideally using a flow diagram. | Results, page 5 |
|  | 16b | Cite studies that might appear to meet the inclusion criteria, but which were excluded, and explain why they were excluded. | Supp.2 (Sheet: included & excluded) |
| Study characteristics | 17 | Cite each included study and present its characteristics. | Supp.2 (All Sheets) |
| Risk of bias in studies | 18 | Present assessments of risk of bias for each included study. | Supp.2 (Sheet:  PROBAST) |
| Results of individual studies | 19 | For all outcomes, present, for each study: (a) summary statistics for each group (where appropriate) and (b) an effect estimate and its precision (e.g. confidence/credible interval), ideally using structured tables or plots. | Supp.2 (Sheet:  CHARMS) |
| Results of syntheses | 20a | For each synthesis, briefly summarise the characteristics and risk of bias among contributing studies. | Supp.2 (Sheet:  PROBAST) |
|  | 20b | Present results of all statistical syntheses conducted. If meta-analysis was done, present for each the summary estimate and its precision (e.g. confidence/credible interval) and measures of statistical heterogeneity. If comparing groups, describe the direction of the effect. | Results, pages 5-9.  No meta-analysis |
|  | 20c | Present results of all investigations of possible causes of heterogeneity among study results. | Discussion, page 11-12 |
|  | 20d | Present results of all sensitivity analyses conducted to assess the robustness of the synthesized results. | Not relevant |
| Reporting biases | 21 | Present assessments of risk of bias due to missing results (arising from reporting biases) for each synthesis assessed. | Not relevant |
| Certainty of evidence | 22 | Present assessments of certainty (or confidence) in the body of evidence for each outcome assessed. | Results, page 9  Supp.2 (Sheets: CHARMS, PROBAST, TRIPOD) |
| **DISCUSSION** | | |  |
| Discussion | 23a | Provide a general interpretation of the results in the context of other evidence. | Discussion, page 9 |
|  | 23b | Discuss any limitations of the evidence included in the review. | Discussion, page 12 |
|  | 23c | Discuss any limitations of the review processes used. | Discussion, page 12 |
|  | 23d | Discuss implications of the results for practice, policy, and future research. | Discussion, page 9-10  Conclusion, page 13 Supp.1. S6 |
| **OTHER INFORMATION** | | |  |
| Registration and protocol | 24a | Provide registration information for the review, including register name and registration number, or state that the review was not registered. | Methods, page 3 |
|  | 24b | Indicate where the review protocol can be accessed, or state that a protocol was not prepared. | Methods, page 3 |
|  | 24c | Describe and explain any amendments to information provided at registration or in the protocol. | - |
| Support | 25 | Describe sources of financial or non-financial support for the review, and the role of the funders or sponsors in the review. | Declared |
| Competing interests | 26 | Declare any competing interests of review authors. | Declared |
| Availability of data, code and other materials | 27 | Report which of the following are publicly available and where they can be found: template data collection forms; data extracted from included studies; data used for all analyses; analytic code; any other materials used in the review. | Methods page 3-4 Supp.2 (All Sheets) |

*From:* Page MJ, McKenzie JE, Bossuyt PM, et al. The PRISMA 2020 statement: an updated guideline for reporting systematic reviews. *BMJ* 2021;372:n71. doi:10.1136/bmj.n71. doi:10.1136/bmj.n71


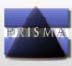
**PRISMA 2020 for Abstracts Checklist**

| **Section and Topic** | **Item #** | **Checklist item** | **Reported (Yes/No)** |
| --- | --- | --- | --- |
| **TITLE** | | |  |
| Title | 1 | Identify the report as a systematic review. | Yes |
| **BACKGROUND** | | |  |
| Objectives | 2 | Provide an explicit statement of the main objective(s) or question(s) the review addresses. | Yes |
| **METHODS** | | |  |
| Eligibility criteria | 3 | Specify the inclusion and exclusion criteria for the review. | Yes |
| Information sources | 4 | Specify the information sources (e.g. databases, registers) used to identify studies and the date when each was last searched. | Yes |
| Risk of bias | 5 | Specify the methods used to assess risk of bias in the included studies. | Yes |
| Synthesis of results | 6 | Specify the methods used to present and synthesise results. | No |
| **RESULTS** | | |  |
| Included studies | 7 | Give the total number of included studies and participants and summarise relevant characteristics of studies. | Yes |
| Synthesis of results | 8 | Present results for main outcomes, preferably indicating the number of included studies and participants for each. If meta-analysis was done, report the summary estimate and confidence/credible interval. If comparing groups, indicate the direction of the effect (i.e. which group is favoured). | Yes |
| **DISCUSSION** | | |  |
| Limitations of evidence | 9 | Provide a brief summary of the limitations of the evidence included in the review (e.g. study risk of bias, inconsistency and imprecision). | Yes |
| Interpretation | 10 | Provide a general interpretation of the results and important implications. | Yes |
| **OTHER** | | |  |
| Funding | 11 | Specify the primary source of funding for the review. | No (reported in “Funding” section) |
| Registration | 12 | Provide the register name and registration number. | No (reported in “Methods” section) |

*From:*  Page MJ, McKenzie JE, Bossuyt PM, Boutron I, Hoffmann TC, Mulrow CD, et al. The PRISMA 2020 statement: an updated guideline for reporting systematic reviews. BMJ 2021;372:n71. doi: 10.1136/bmj.n71

| ***Criterium*** | ***Inclusions*** | ***Exclusions*** |
| --- | --- | --- |
| 1. *Population* | - Adults - Elderly - Age is unspecified, and the population contains mainly adults. - Subgroup populations (home care, veteran) | - Pediatrics - Specific disease population - Admission specific to surgical, rehabilitation, newborn, and other elective procedures |
| 1. *Admission* | - Admission and readmission from outside hospital (ex. home, home healthcare, other facilities) - Admission readmission from ED to wards - Admissions and readmissions from ED to ICU - Admissions form ambulance to hospital admission - ICU readmissions - All-cause admissions - Somatic related hospitalizations | - In-hospital admission (from ICU to wards for example, or from one department to another) - Admission to ED only (we don’t consider ED room as a part of the admission process as the patient can be discharged from ED) - ED revisits - ED boarding - ED demand - Disease-specific admissions/readmissions - Psychiatric related hospitalization |
| 1. *Methods* | - Different types of machine learning - LR is only included if it is included in a machine learning context. | - Traditional statistical prediction using LR, multivariable LR, Cox, etc. that are not a part of a machine learning methodology. - Risk score indexes (score) which is not in a machine learning context. |
| 1. *Data* | - Real world datasets | - Simulated, synthetic datasets |
| 1. *Research* | - Original research - English | - Reviews - Reports - Comments - Studies/abstract with incomplete/low-quality of reporting - Duplicated studies/abstracts with full-text articles |

# 2. Inclusions and exclusions criteria

*Table S2.1 The systematic review inclusion/exclusion criteria*


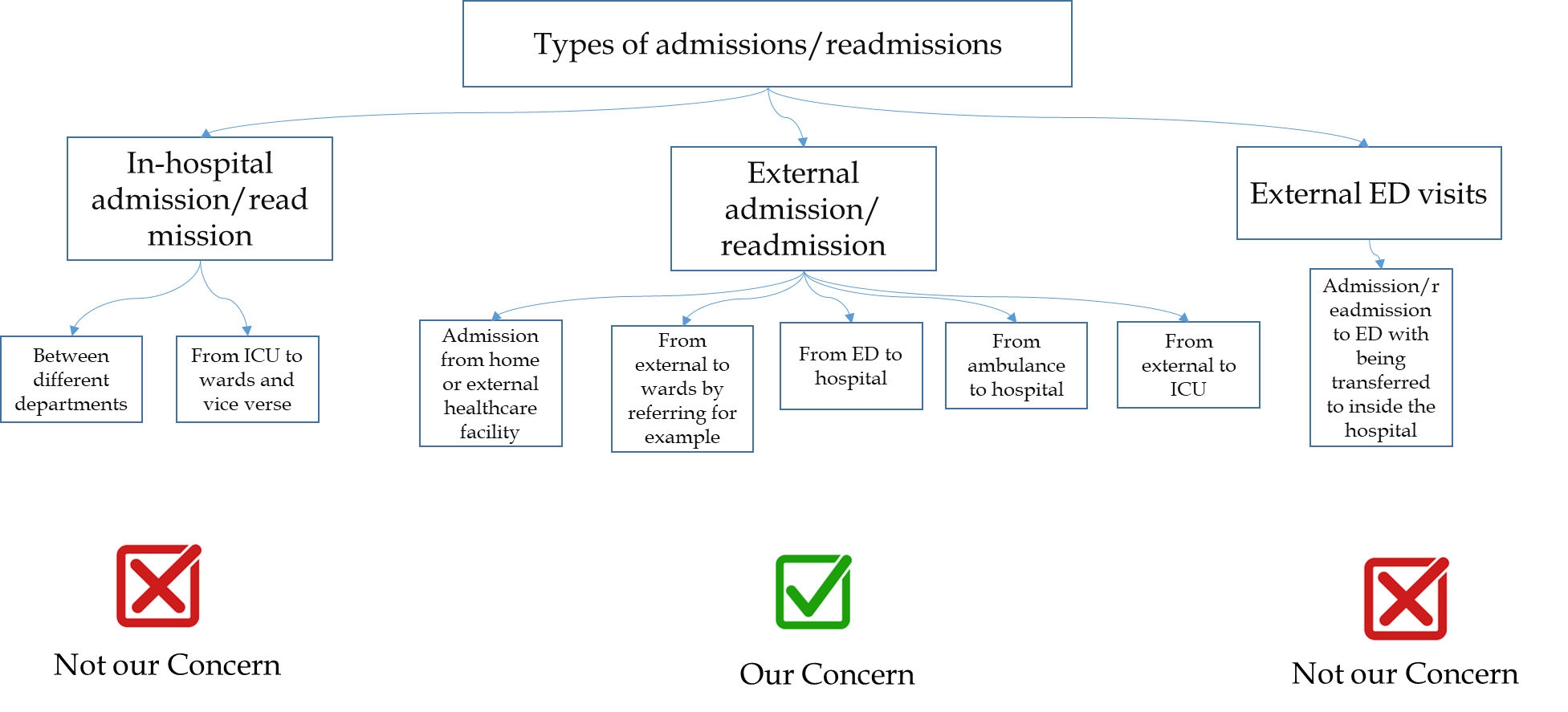


*Figure S 1.1 Defining admissions/readmissions in the review.*

3. Literature search syntax **(search date: 17.10.2023)**

1. **Main databases**

| **Category** | **Search syntax** | **# Articles** |
| --- | --- | --- |
| 1. **PubMed** | | |
| **Prediction**  **#1** | (((predict* AND clinical OR outcome* OR risk* OR model*) OR (validat* AND model* OR rule* OR algorithm*) OR (prognos* AND model OR risk* OR rule*) OR ((identify* OR classif* OR discriminat* OR estimate*) AND risk* OR model* OR outcome*)) OR ((compar* OR develop*) AND model* OR machine learning OR algorithm* OR tool) AND (AUC OR c statistic OR c-statistic OR ROC OR accuracy)) | **585,836** |
| **Hospitalization**  **#2** | ("Hospitalization"[MeSH] OR hospitali* [Title/Abstract] OR "Inpatients"[MeSH] OR inpatient [Title/Abstract] OR "Admitting Department, Hospital"[MeSH] OR admis*[Title/Abstract] OR readmission*[Title/Abstract]) | **804,747** |
| **Machine learning**  **#3** | (("Artificial Intelligence"[Mesh] OR "Support Vector Machine"[Mesh] OR "Deep Learning"[Mesh] OR Machine Learning OR Artificial Intelligence OR Naive Bayes OR bayesian learning OR Neural Network* OR K nearest neighbor* OR K-nearest neighbor* OR Decision Trees OR Natural Language Processing OR support vector* OR random forest OR boosting OR deep learning OR machine intelligence OR computational intelligence OR computer reasoning OR Algorithm)) | **1,081,677** |
| **Exclusions**  **(Specified diseases’ research, younger pop., irrelevant ML research)**  **#4** | ((((image[Title/Abstract] OR imaging[Title/Abstract] OR MRI[Title/Abstract] OR magnetic resonance [Title/Abstract] OR electrocardiogram[Title/Abstract] OR ECG[Title/Abstract] OR EEG[Title/Abstract] OR signal*[Title/Abstract] OR ultrasound[Title/Abstract] OR angle*[Title/Abstract] OR genet*[Title/Abstract] OR COVID [Title/Abstract] OR handwrit*[Title/Abstract] OR signature[Title/Abstract] OR device OR robo* OR smartwatch OR smartphone OR sensor* OR wear* OR delirium[Title] OR cancer[Title] OR cardiac[Title] OR heart[Title] OR surgery [Title] OR schizophrenia[Title] OR bacter*[Title] OR antibiotic*[Title] OR asthma[Title] OR psych*[Title] OR infection*[Title] OR sepsis[Title] OR organ[Title] OR hemorrhage[Title] OR hypertens*[Title] OR hypotens[Title] OR myocardial[Title] OR cardio*[Title] OR trauma*[Title] OR syndrome[Title] OR insulin[Title] OR diabet*[Title] OR transplant*[Title] OR kidney[Title] OR renal[Title] OR pancreat*[Title] OR ulcer[Title] OR depress*[Title] OR urinary[Title] OR COPD[Title] OR procalcitonin[Title] OR nutrition[Title] OR sarcopenia[Title] OR pneumonia[Title] OR burn[Title] OR stroke[Title] OR blood[Title] OR arthritis[Title] OR dementia[Title] OR vascular[Title] OR radio*[Title] OR fall*[Title] OR pulmonary[Title] OR serum[Title] OR thromb*[Title] OR wound[Title] OR ventricular[Title] OR lung[Title] OR liver[Title] OR hip[Title] OR fibrillation[Title] OR neuro*[Title] OR respiratory[Title] OR mental[Title] OR artery[Title] OR aortic[Title] OR influenza[Title] OR HIV[Title] OR parkinson*[Title] OR cognitive[Title] OR spinal[Title] OR knee[Title] OR sclerosis[Title] OR gastro*[Title] OR bipolar[Title] OR tuberculosis[Title] OR bowel[Title] OR opioid[Title] OR fracture[Title] OR suicide[Title] OR fever[Title] OR hepatitis[Title] OR staph*[Title] OR intravenous[Title] OR child*[Title] OR pediat*[Title] OR neonat*[Title] OR infant*[Title])))) | **17,567,542** |
| **Combining** | #1 AND #2 AND #3 NOT #4 | **1,077** |
| 1. **Web of Science** | | |
| **Prediction**  **#1** | TS=((((((predict* AND clinical OR outcome* OR risk* OR model* ) OR (validat* AND model* OR rule* OR algorithm* ) OR (prognos* AND model OR risk* OR rule* ) OR ((identify* OR classif* OR discriminat* OR estimate* ) AND risk* OR model* OR outcome* )) OR ((compar* OR develop*) AND model* OR "machine learning" OR algorithm* OR tool ) AND (AUC OR "c statistic" OR c-statistic OR ROC OR accuracy ))))) | **18,954,045** |
| **Hospitalization**  **#2** | TS=((Hospitalization OR hospitali* OR Inpatients OR inpatient OR "Admitting Department, Hospital" OR admis* OR readmission*)) | **721,490** |
| **Machine learning**  **#3** | TS=(("Artificial Intelligence" OR "Support Vector Machine" OR "Deep Learning" OR "Machine Learning" OR "Artificial Intelligence" OR "Naive Bayes" OR "bayesian learning" OR "Neural Network*" OR "K nearest neighbor*" OR "K-nearest neighbor*" OR "Decision Trees" OR "Natural Language Processing" OR "support vector*" OR "random forest" OR boosting OR "deep learning" OR "machine intelligence" OR "computational intelligence" OR "computer reasoning" OR Algorithm )) | **3,584,025** |
| **Exclusions**  **(Specified diseases’ research, younger pop., irrelevant ML research)**  **#4** | TS=((((image OR imaging OR MRI OR "magnetic resonance" OR electrocardiogram OR ECG OR EEG OR signal* OR ultrasound OR angle* OR genet* OR COVID OR handwrit* OR signature OR device OR robo* OR smartwatch OR smartphone OR sensor* OR wear* OR delirium OR cancer OR cardiac OR heart OR surgery OR schizophrenia OR bacter* OR antibiotic* OR asthma OR psych* OR infection* OR sepsis OR organ OR hemorrhage OR hypertens* OR hypotens OR myocardial OR cardio* OR trauma* OR syndrome OR insulin OR diabet* OR transplant* OR kidney OR renal OR pancreat* OR ulcer OR depress* OR urinary OR COPD OR procalcitonin OR nutrition OR sarcopenia OR pneumonia OR burn OR stroke OR blood OR arthritis OR dementia OR vascular OR radio* OR fall* OR pulmonary OR serum OR thromb* OR wound OR ventricular OR lung OR liver OR hip OR fibrillation OR neuro* OR respiratory OR mental OR artery OR aortic OR influenza OR HIV OR parkinson* OR cognitive OR spinal OR knee OR sclerosis OR gastro* OR bipolar OR tuberculosis OR bowel OR opioid OR fracture OR suicide OR fever OR hepatitis OR staph* OR intravenous OR child* OR pediat* OR neonat* OR infant*)))) | **32,988,115** |
| **Combining** | (((#1) AND #2) AND #3) NOT #4 | **7,625** |
| **Including relevant fields** | ((((#1) AND #2) AND #3) NOT #4) AND (LA==("ENGLISH") AND TASCA==("MEDICAL INFORMATICS" OR "HEALTH CARE SCIENCES SERVICES" OR "COMPUTER SCIENCE INTERDISCIPLINARY APPLICATIONS" OR "MEDICINE GENERAL INTERNAL" OR "PUBLIC ENVIRONMENTAL OCCUPATIONAL HEALTH" OR "PHARMACOLOGY PHARMACY" OR "MULTIDISCIPLINARY SCIENCES" OR "HEALTH POLICY SERVICES" OR "EMERGENCY MEDICINE" OR "CRITICAL CARE MEDICINE" OR "MEDICINE RESEARCH EXPERIMENTAL" OR "PRIMARY HEALTH CARE")) | **1,271** |
| 1. **CINAHL via EBSCOhost** | | |
| **Prediction**  **#1** | (((((predict* AND clinical OR outcome* OR risk* OR model* ) OR (validat* AND model* OR rule* OR algorithm* ) OR (prognos* AND model OR risk* OR rule* ) OR ((identify* OR classif* OR discriminat* OR estimate* ) AND risk* OR model* OR outcome* )) OR ((compar* OR develop*) AND model* OR "machine learning" OR algorithm* OR tool) AND (AUC OR "c statistic" OR c-statistic OR ROC OR accuracy )))) | **Combined search**  **2,256** |
| **Hospitalization**  **#2** | ((MH Hospitalization+) OR (TI hospitali* OR AB hospitali*) OR (MH Inpatients+) OR (TI inpatient OR AB inpatient) OR (MH "Admitting Department, Hospital"+) OR (TI admis* OR AB admis*) OR (TI readmission* OR AB readmission*)) |  |
| **Machine learning**  **#3** | ((MH "Machine Learning"+) OR (MH "Artificial Intelligence"+) OR (MH "Natural Language Processing"+) OR (MH "Neural Networks, Computer"+) OR (MH "Support Vector Machine"+) OR (MH "Deep Learning"+) OR (MH "Regression Analysis"+) OR "Machine Learning" OR "Artificial Intelligence" OR "Naive Bayes" OR "bayesian learning" OR "Neural Network*" OR "K nearest neighbor*" OR "K-nearest neighbor*" OR "Decision Trees" OR "Natural Language Processing" OR "support vector*" OR "random forest" OR (TI boosting OR AB boosting) OR "deep learning" OR "machine intelligence" OR "computational intelligence" OR "computer reasoning" OR Algorithm) |  |
| **Exclusions**  **(Specified diseases’ research, younger pop., irrelevant ML research)**  **#4** | (((((TI image OR AB image) OR (TI imaging OR AB imaging) OR (TI MRI OR AB MRI) OR (TI "magnetic resonance" OR AB "magnetic resonance") OR (TI electrocardiogram OR AB electrocardiogram) OR (TI ECG OR AB ECG) OR (TI EEG OR AB EEG) OR (TI signal* OR AB signal*) OR (TI ultrasound OR AB ultrasound) OR (TI angle* OR AB angle*) OR (TI genet* OR AB genet*) OR (TI COVID OR AB COVID) OR (TI handwrit* OR AB handwrit*) OR (TI signature OR AB signature) OR device OR robo* OR smartwatch OR smartphone OR sensor* OR wear* OR TI delirium OR TI cancer OR TI cardiac OR TI heart OR TI surgery OR TI schizophrenia OR TI bacter* OR TI antibiotic* OR TI asthma OR TI psych* OR TI infection* OR TI sepsis OR TI organ OR TI hemorrhage OR TI hypertens* OR TI hypotens OR TI myocardial OR TI cardio* OR TI trauma* OR TI syndrome OR TI insulin OR TI diabet* OR TI transplant* OR TI kidney OR TI renal OR TI pancreat* OR TI ulcer OR TI depress* OR TI urinary OR TI COPD OR TI procalcitonin OR TI nutrition OR TI sarcopenia OR TI pneumonia OR TI burn OR TI stroke OR TI blood OR TI arthritis OR TI dementia OR TI vascular OR TI radio* OR TI fall* OR TI pulmonary OR TI serum OR TI thromb* OR TI wound OR TI ventricular OR TI lung OR TI liver OR TI hip OR TI fibrillation OR TI neuro* OR TI respiratory OR TI mental OR TI artery OR TI aortic OR TI influenza OR TI HIV OR TI parkinson* OR TI cognitive OR TI spinal OR TI knee OR TI sclerosis OR TI gastro* OR TI bipolar OR TI tuberculosis OR TI bowel OR TI opioid OR TI fracture OR TI suicide OR TI fever OR TI hepatitis OR TI staph* OR TI intravenous OR TI child* OR TI pediat* OR TI neonat* OR TI infant*)))) |  |
| **Combining** | (((#1) AND #2) AND #3) NOT #4 limit to English | **2,203** |
| 1. **Embase via Ovid** | | |
| **Prediction**  **#1** | (((((predict* AND clinical OR outcome* OR risk* OR model* ) OR (validat* AND model* OR rule* OR algorithm* ) OR (prognos* AND model OR risk* OR rule* ) OR ((identify* OR classif* OR discriminat* OR estimate* ) AND risk* OR model* OR outcome* )) OR ((compar* OR develop* ) AND model* OR "machine learning" OR algorithm* OR tool) AND (AUC OR "c statistic" OR c-statistic OR ROC OR accuracy)))) | **763,454** |
| **Hospitalization**  **#2** | (exp Hospitalization/ OR hospitali*.tw. OR exp Inpatients/ OR inpatient.tw. OR exp "Admitting Department, Hospital"/ OR admis*.tw. OR readmission*.tw.) | **153,6555** |
| **Machine learning**  **#3** | exp "Machine Learning"/ or exp "Artificial Intelligence"/ or exp "Natural Language Processing"/ or exp "artificial neural network "/ or exp "Support Vector Machine"/ or exp "Deep Learning"/ or exp "Regression Analysis"/ or "Machine Learning".mp. or "Artificial Intelligence".mp. or "Naive Bayes".mp. or "bayesian learning".mp. or "Neural Network*".mp. or "K nearest neighbor*".mp. or "K-nearest neighbor*".mp. or "Decision Trees".mp. or "Natural Language Processing".mp. or "support vector*".mp. or "random forest".mp. or boosting.tw. or "deep learning".mp. or "machine intelligence".mp. or "computational intelligence".mp. or "computer reasoning".mp. or Algorithm.mp. | **815,794** |
| **Exclusions**  **(Specified diseases’ research, younger pop., irrelevant ML research)**  **#4** | ((((image.tw. OR imaging.tw. OR MRI.tw. OR "magnetic resonance".tw. OR electrocardiogram.tw. OR ECG.tw. OR EEG.tw. OR signal*.tw. OR ultrasound.tw. OR angle*.tw. OR genet*.tw. OR COVID.tw. OR handwrit*.tw. OR signature.tw. OR device OR robo* OR smartwatch OR smartphone OR sensor* OR wear* OR delirium.ti. OR cancer.ti. OR cardiac.ti. OR heart.ti. OR surgery.ti. OR schizophrenia.ti. OR bacter*.ti. OR antibiotic*.ti. OR asthma.ti. OR psych*.ti. OR infection*.ti. OR sepsis.ti. OR organ.ti. OR hemorrhage.ti. OR hypertens*.ti. OR hypotens.ti. OR myocardial.ti. OR cardio*.ti. OR trauma*.ti. OR syndrome.ti. OR insulin.ti. OR diabet*.ti. OR transplant*.ti. OR kidney.ti. OR renal.ti. OR pancreat*.ti. OR ulcer.ti. OR depress*.ti. OR urinary.ti. OR COPD.ti. OR procalcitonin.ti. OR nutrition.ti. OR sarcopenia.ti. OR pneumonia.ti. OR burn.ti. OR stroke.ti. OR blood.ti. OR arthritis.ti. OR dementia.ti. OR vascular.ti. OR radio*.ti. OR fall*.ti. OR pulmonary.ti. OR serum.ti. OR thromb*.ti. OR wound.ti. OR ventricular.ti. OR lung.ti. OR liver.ti. OR hip.ti. OR fibrillation.ti. OR neuro*.ti. OR respiratory.ti. OR mental.ti. OR artery.ti. OR aortic.ti. OR influenza.ti. OR HIV.ti. OR parkinson*.ti. OR cognitive.ti. OR spinal.ti. OR knee.ti. OR sclerosis.ti. OR gastro*.ti. OR bipolar.ti. OR tuberculosis.ti. OR bowel.ti. OR opioid.ti. OR fracture.ti. OR suicide.ti. OR fever.ti. OR hepatitis.ti. OR staph*.ti. OR intravenous.ti. OR child*.ti. OR pediat*.ti. OR neonat*.ti. OR infant*.ti.)))) | **13,229,299** |
| **Combining**  **#5** | ((1 and 2 and 3) not 4) | **1,926** |
|  | limit 6 to english language | **1,873** |

**2. Grey literature search (15.11.2021)**

| **OpenGrey.eu** | | |
| --- | --- | --- |
| Search syntax | (predict* OR prognos* OR validat*) AND (hospit* OR *admission*) AND (machine* OR intelligence) (English) | **13** |
| **MedNar.com** | | |
| Search syntax | ti:(predict* OR prognos* OR validat*) AND (hospit* OR *admission*) AND (machine* OR intelligence) | **327** |
| **WorldCat(OCLC)** | | |
| Search syntax | ((ti: predict* OR ti: prognos* OR ti: validat*)) and ((ti: machine* OR ti: intelligence)) and ((ti: hospit* OR ti: *admission*)) and la= "eng" | **230** |
| **ProQuest** |  |  |
| Search syntax | ((AB,TI(predict* OR prognos* OR validat*) AND AB,TI(hospit* OR admission* OR readmission OR re-admission) AND AB,TI(machine* OR intelligence)) AND (at.exact(("Conference Proceeding" OR "Dissertation/Thesis" OR "Undefined" OR "Working Paper/Pre-Print" OR "News" OR "Review" OR "Report" OR "Commentary" OR "Conference" OR "Book" OR "Conference Paper") NOT ("Article" OR "Feature")) AND la.exact("ENG") AND subt.exact(("machine learning" OR "artificial intelligence" OR "algorithms" OR "patients" OR "neural networks" OR "support vector machines" OR "prediction models" OR "health care" OR "electronic health records" OR "decision trees" OR "mathematical models" OR "classification" OR "hospitals" OR "artificial neural networks" OR "data sets" OR "decision making" OR "mortality" OR "health care management" OR "data mining" OR "predictions" OR "accuracy" OR "public health" OR "deep learning" OR "physicians" OR "bioinformatics" OR "feature extraction" OR "model accuracy" OR "doctors" OR "classifiers" OR "datasets" OR "regression analysis" OR "learning algorithms" OR "medicine" OR "optimization" OR "risk analysis" OR "training" OR "emergency medical services" OR "diagnostic systems" OR "data analysis" OR "decision support systems" OR "nursing" OR "statistical analysis" OR "automation" OR "performance prediction" OR "regression models" OR "bayesian analysis" OR "signs and symptoms" OR "intensive care" OR "software" OR "risk" OR "information technology" OR "big data" OR "medical research" OR "natural language processing" OR "data management" OR "health sciences" OR "health services" OR "patient admissions" OR "medical personnel" OR "epidemiology" OR "risk assessment" OR "data collection" OR "performance evaluation") NOT ("computer science" OR "covid-19" OR "coronaviruses" OR "medical imaging" OR "diagnosis" OR "psychotherapy" OR "colleges & universities" OR "industrial engineering" OR "diabetes" OR "biomedical engineering" OR "sepsis" OR "students" OR "surgery" OR "viral diseases" OR "computer simulation" OR "cancer" OR "cardiovascular disease" OR "heart" OR "higher education" OR "engineering" OR "signal processing" OR "heart diseases" OR "heart failure" OR "educational psychology" OR "internet of things" OR "magnetic resonance imaging" OR "cloud computing" OR "stroke" OR "applications programs" OR "cardiology" OR "education" OR "electrical engineering" OR "electroencephalography" OR "medical equipment" OR "mental health" OR "pandemics" OR "stock exchanges")))) AND subt.exact("machine learning") | **549** |

# 4. Data sources and their frequency of use in the included studies

***Data collection considering time perspective***

From the data collecting time perspective, sources of data used can be divided into 4 main categories; 1) preadmission data sources such as General Practitioner centers (GP centers) [1], Home healthcare data [2], call centers’ data [3], 2) upon admission sources such as data from ambulances [4], ED and triage data [5–7], and dispatch centers [8], 3) data collected under admission such as data from EHR [9–11], ICU [12–14], and hospital information systems [15], 4) data collected in health registers [16], and claim registers [17–19]. Figure S4.1, S4.2 summarizes the sources of data used in the articles included in this review.


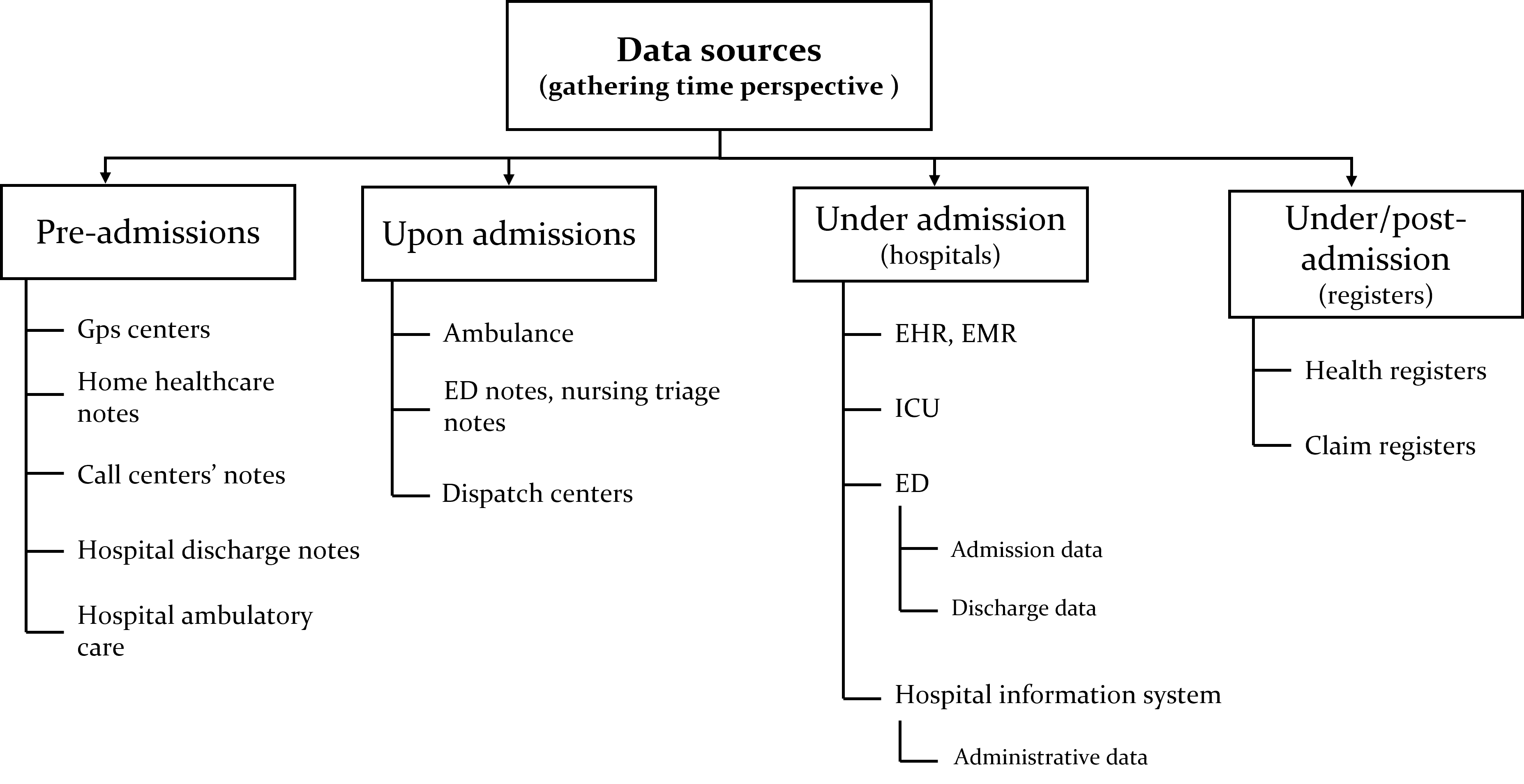
*Figure S4.1. Sources of data used in the included studies from the time of gathering perspective.*

*Table S4.1. The types and sources of data sorted by the predicted outcome. Darker cells indicate more studies.* *ED data was mostly used to predict admissions, while EHR, EMR, ICU data was mostly used to predict readmissions.*

| **Data sources** | | **Predicting admission** | **Predicting readmission** | **Total** |
| --- | --- | --- | --- | --- |
| **Hospital** | **Hospital** | 0 | 14 | 14 |
|  | **Hospital (administrative)** | 4 | 6 | 10 |
|  | **Hospital (ambulatory)** | 3 | 0 | 3 |
| **Medical records** | **ED** | 23 | 1 | 24 |
|  | **EHR** | 7 | 19 | 26 |
|  | **EHR+ED** | 1 | 0 | 1 |
|  | **EMR** | 1 | 6 | 7 |
|  | **ICU** | 0 | 18 | 18 |
|  | **GP centers** | 1 | 0 | 1 |
| **Registers** | **Register** | 0 | 1 | 1 |
|  | **Register (claim)** | 1 | 5 | 6 |
| **Related medical notes** | **Call center notes** | 1 | 0 | 1 |
|  | **Discharge notes** | 0 | 1 | 1 |
|  | **Home healthcare clinical notes** | 2 | 0 | 2 |
| **Ambulance/ Dispatch** | **Ambulance** | 1 | 0 | 1 |
|  | **Ambulance + dispatch** | 1 | 0 | 1 |
|  | **Total** | 46 | 71 | **117*** |

- ***Abbreviations****: ED: Emergency Department; HER: Electronic Health Records; EMR: Electronic Medical Records; ICU: Intensive Care Unit; GP: General Practitioner. Total number of sources is 117 as there is one study predicted both hospital admissions and readmissions.*

** One study investigated both admission and readmission.*

*Figure S4.2. Sources of data used for predicting hospital admission and readmission.*

- ***Abbreviations****: ED: Emergency Department; HER: Electronic Health Records; EMR: Electronic Medical Records; ICU: Intensive Care Unit; GP: General Practitioner.*

# 5. Benchmarking with risk indexes

In total 18 studies benchmarked their model against risk indexes. Fourteen different risk indexes/scores were used for benchmarking namely LACE [20], PARR [21], CMS model for hospital profiling [22], APACHE II [23], III [24], ESI [25], NEWS [26], CPM [27], START [28], Charlson Index[29], HOSPITAL [30], LACE-rt [31], SAPS II [32], HLCC [33]v. Figure S7.1 represents the number of studies that used each risk index.

Studies’ models [11,34–41] were benchmarked against LACE, [34,42] against PARR, studies [11,35] models against HOSPITAL, [43] against START, [44] against ESI index, [45] against APACHE II, III, LACE-rt [11], NEWS [8], and [46] against CPM, [47] against CMS, [48] against Charlson index, [49] against SAPS, and [50] against HLCC.

*Figure S7.1. The number of studies that used risk indexes in benchmarking ML models. Some studies benchmarked their models against more than one risk index.*

- ***Abbreviations:*** ***LACE****: An index for assessing the risk of 30-day readmission;* ***PARR****: Patients At Risk of Hospital Readmission Index;* ***CMS****: The Centers for Medicare & Medicaid Services model for hospital profiling;* ***APACHE****:* *Acute Physiology and Chronic Health Evaluation index,* ***ESI****:* *Emergency Severity Index;* ***NEWS****:* *National Early Warning Scores;* ***CPM****: Combined Predictive Model index;* ***START****: Sydney Triage Admission Risk Tool;* ***HOSPITAL****: A score for assessing the risk of 30-day readmission,* ***SAPS*** *II: Simplified Acute Physiology Score,* ***HLCC****: HealthLinks Chronic Care Program.*

# 6. On the generalizability of ML models

Akin to external validity, the generalizability of ML models refers to the model being able to perform similarly on external independent patient cohorts [51,52]. While external validity and generalizability terms are used interchangeably, external validity is a wider term and often refers to two underlying concepts, generalizability, and applicability [53]. Here, we refer to generalizability as a step prior to external validity and involves steps of acquiring and harmonizing datasets before implementing the trained models.

In general, there are three levels of implementing ML models: i) implementing on the institutional level and this is the approach adopted by the majority of ML studies. This approach is arguably the realistic approach to follow at the moment in light of the present external validity limitations [39] [54]. The approach can be extended to include multiple institutions with a Common Data Model (CDM) between them [55] ii) implementing models on the national level which is possible if adopted by policymakers and the needed IT infrastructure is there. An example of implementing ML models on the national level is the Business Research Analytics Insights Network (BRAIN) system in Singapore [56]. The system utilizes daily data from multiple sources and provides near real-time healthcare risk predictions [57]. iii) Implementing on an international level is the hope but still considerably far away from achieving due to many obstacles. These can be summarized in two main criteria: i) model-related, and ii) data-related obstacles. Model-related obstacles include being transparent about developing the model as mentioned in the reporting quality and reproducibility subsection. The data-related obstacles include the wide variety of data structure formats, different preprocessing steps, different data distributions, population differences, and restricted data privacy regulations.

Individual solutions exist for most of these problems. Adopting acceptable CDMs across similar institutions to standardize the structure of healthcare datasets could be a solution for different data formats which hinder easily implementing models on different datasets. Standardizing data preprocessing steps can be challenging but still required to harmonize the different datasets. Dataset similarity metrics [58,59] can also be used as a partial solution to determine if the data distributions between the different datasets are dissimilar to give a signal if the model will perform poorly on the external datasets.

Federated learning (FL) is a learning technique that aims to train ML algorithms collaboratively on several datasets without moving the datasets themselves from their original servers or machines [60]. The approach solves the problem of data privacy as it does not include sharing the data but only the final predictions [61]. Still, the approach has some limitations, and the system has to be designed properly to address privacy and security hazards [62,63]. Yet, adopting a stepwise road map that incorporates all these solutions to tackle the problem doesn’t exist. Optimistically, it has now become more obvious to researchers that it is time to shift focus from model development to model deployment and from models to data [64].

# 7. A suggestion for a reporting checklist for ML models in structured datasets

| 1. **Title** | |
| --- | --- |
|  | Identify the main domains in the title |
| 1. **Abstract** | |
|  | Include background, objective, study type, data source, data time frame, population methods: models, performance metrics with CI, conclusion |
| 1. **Introduction** | |
|  | Include the main concepts and definitions, related work, objectives, study type, and rationale for performing the study |
| 1. **Methods and results** | |
| 1. ***Data*** | |
| Data sources, time frame | Specify the methods of data collection or acquisition. What is the source of data (hospital, register, RCTs, others)? |
| Data type | What types of datasets were used (clinical, administrative, both)? |
| ***a.1) Population*** | |
| Characteristics, Description | Describe the population characteristics |
| Eligibility criteria | Describe the eligibility criteria to be included in the study |
| Cohort size | What was the size of the included cohort size? |
| ***a.2) Exploratory Data Analysis (EDA)*** | |
| Univariate analysis | Include descriptive analysis for each variable (mean, type, missing, cardinality. etc.) |
| Bivariate analysis | Investigate the pairwise correlation and interactions between the included features |
| Duplicates | Were duplicates investigated? |
| Outliers | Were outliers investigated? |
| Differences between training and validation cohorts | Describe the training and validation datasets. Report if there were differences between training and validating sets. Did you provide data similarity metrics? |
| ***a.3) Outcomes*** | |
|  | Provide an explicit description of the outcome type and definition. |
| 1. ***Data preprocessing*** | |
| ***b.1) Missing data handling*** | |
| Reporting missing values | Include percent in each feature, overall missing percent |
| Method(s) of handling | Describe how missing values were handled. |
| ***b.2) Data balance*** | |
| Report data balance/imbalance | Report the outcome class(es) percent. If the classes were imbalanced, was there a way to acquire more data to balance them? |
| Method(s) of handling | If not, what was the method used to balance the classes? Does this method have drawbacks? What are they? |
| ***b.3) Outlier’s handling*** | |
| Report outliers | Were the dataset outliers reported? |
| Method(s) of handling | How were they handled? |
| ***b.4) Correlation and interactions (bivariate analysis) handling*** | |
|  | In case of strong correlation between features, how was that handled? |
| ***b.5) Categorical features handling*** | |
| Encoding method(s) | Does the ML algorithm require handling categorical features? What encoding method was used? |
| Grouping method(s) | In the case of high-dimensional features (e.g., hierarchal code systems), what grouping/truncation methods were used? |
| High-cardinality variables handling | Were high-cardinality variables investigated? Describe how they were handled. |
| ***b.6) Continuous features handling*** | |
|  | Does the ML algorithm require handling continuous features? What method was used (normalization, standardization, etc.)? |
| ***b.7) Time-series features handling*** | |
|  | Describe how time-series features were handled. |
| ***b.8) Longitudinal data (multiple rows id) handling*** | |
|  | How was longitudinal data handled? |
| ***b.9) Generated/transformed features*** | |
|  | Were new features generated/introduced to the original dataset? What are they? What reason behind introducing them? |
| 1. ***Feature analysis*** | |
| *No. of features for the initial and final model* | Report the total number of features in the original dataset and the features used in the model. |
| *Feature selection methods* | What methods were used for feature selection? |
| *Feature selection validated with other studies/experts etc.* | Were the selected features validated with similar studies or domain experts? |
| 1. ***Model development*** | |
| *Data splitting* | Which method(s) was used for dataset splitting? |
| *Assess sufficient observations for a good fit of the model* | Were there sufficient data instances (records) for training the models? |
| *ML algorithms used* | Which ML algorithms were used? |
| *Satisfying models’ assumptions* | Were the algorithms' assumptions investigated and satisfied? |
| *Bases of model(s) choice* | What were the reasons behind using these specific algorithms? |
| *Hyperparameter tuning* | Was hyperparameter tuning performed? How was it done, and what were the results? |
| *Model results specifications* | What were the results regarding model specifications (ex. regression coefficients, OR, etc.,)? |
| *Data threshold gives the best results* | Was the amount threshold of which the dataset gives the best performance investigated? |
| *Time of training* | How long did the training time take for each algorithm? |
| *Risk of information leakage assessed* | Has the risk of information leak been considered in developing the model? What has been done to avoid that? |
| 1. ***Model validation*** | |
| *Internal model validation* | What methods of internal validation were used? |
| *External validation* | Was the model externally validated? which method(s) was used? |
| *Prospective validation* | Was the model prospectively validated? |
| *Benchmarking with a risk index/score* | Was there a validated or well-known risk score/index in the domain? Benchmark the developed models against them. |
| *Compare with a baseline model* | If no validated risk indexes, search the literature for baseline models in the domain and compare the model with them. |
| 1. ***Evaluation*** | |
| *Evaluation metric for model performance (e.g., AUC, acc..)* | Report what evaluation metrics were used. |
| *Evaluation metric for clinical performance (e.g., Sensitivity, PPV, NPV)* | In case of predicting a health-related outcome, provide clinical performance metrics and report them. |
| *Bases of evaluation metrics choice* | The reasons behind selecting specific evaluation metrics |
| *Best performing model* | Report the best-performing model. |
| *Generalizability* | Elaborate on the generalizability of the model to other populations/datasets. |
| *Significance difference in results/test* | Report if the difference between models’ performance is significant. What was the statistical test used? |
| *Confidence intervals* | Report 95% CI. Describe the method used for calculating the CI. |
| 1. ***Calibration*** | |
| *Model calibrated* | Provide a calibration method for the model predictions. |
| *Method of calibration* | Which calibration method has been used? |
| 1. ***Interpretation*** | |
| *Global interpretation* | Did the study provide global interpretations of the model outputs? What are they? |
| *Local interpretation* | Did the study provide local interpretations of the model outputs? What are they? |
| *Explaining a sample of wrong predictions* | If the study provided local interpretations, can the model’s incorrect predictions be explained? Explain with some examples. |
| 1. ***Deployment in settings*** | |
| *Methods of deploying* | Was the model deployed in practice? Explain the method of deployment. |
| *Updating model frequency* | How frequently is the model training undertaken? |
| *Model performance changes over time* | After the training update, provide the new model performance. |
| 1. ***Flow diagram of model-developing steps (recommended)*** | |
|  | If building, validating, or deploying the models are multistep, provide a flow diagram of these steps. |
| 1. ***Software/Hardware*** | |
| *Software specifications* | Specify which software/package versions were used in developing the model. |
| *Hardware specifications* | Provide the specifications of the hardware used to develop the model(s). |
| 1. ***Discussion*** | |
| *General* | Provide a general interpretation of the results, and implications of the results for practice, policy, and future research. |
| *Limitations* | Report model, design, and study limitations |
| *Possible implications* | What are the implications of the developed model? |
| *Assess the risk of overfitting* | Was there a risk of overfitting because of the algorithm or the development design |
| *Assess the risk of poor data quality* | Was the data quality assessed? Will this affect the results? |
| *Assess the risk of systemic bias in the dataset.* | Was the risk of systematic bias in the dataset assessed? |
| 1. ***Conclusion*** | |
|  | Provide a concise conclusion of the findings and implications. |
| 1. ***Supporting information*** | |
| *Dataset availability* | Is the dataset publicly available/upon request/not available? |
| *Code availability* | Provide the code used in developing the model. |
| *Funding* | Provide funding/competing interest information. |
| *Supplementary* | Data quality check: Is checking data quality relevant for the research dataset? If so, what were the dimensions checked (e.g., correctness, completeness, timeliness, concordance, uniqueness, etc.,)? What were the methods used in assessing these dimensions?  Provide any necessary or relevant information that couldn’t be provided in the manuscript. |

- *This checklist is not validated and serves only as a suggestion for a reporting checklist for Machine Learning models predicting health-related outcomes in structured datasets.*

# References

1. Khanna S, Rolls DA, Boyle J, Xie Y, Jayasena R, Hibbert M, et al. A risk stratification tool for hospitalisation in Australia using primary care data. Sci Rep. 2019;9. doi:10.1038/s41598-019-41383-y

2. Topaz M, Woo K, Ryvicker M, Zolnoori M, Cato K. Home Healthcare Clinical Notes Predict Patient Hospitalization and Emergency Department Visits. Nurs Res. 2020;69: 448–454. doi:10.1097/NNR.0000000000000470

3. Lorenzana A, Tyagi M, Wang QC, Chawla R, Nigam S. Using text notes from call center data to predict hospitalization. Value Heal. 2016;19: A87. Available: http://ovidsp.ovid.com/ovidweb.cgi?T=JS&PAGE=reference&D=emed17&NEWS=N&AN=72311155

4. Shirakawa T, Sonoo T, Ogura K, Fujimori R, Hara K, Goto T, et al. Institution-Specific Machine Learning Models for Prehospital Assessment to Predict Hospital Admission: Prediction Model Development Study. JMIR Med INFORMATICS. 2020;8. doi:10.2196/20324

5. Peck JS, Benneyan JC, Nightingale DJ, Gaehde SA. Predicting emergency department inpatient admissions to improve same-day patient flow. Acad Emerg Med Off J Soc Acad Emerg Med. 2012;19: E1045-54. doi:10.1111/j.1553-2712.2012.01435.x

6. Mowbray F, Zargoush M, Jones A, de Wit K, Costa A. Predicting hospital admission for older emergency department patients: Insights from machine learning. Int J Med Inform. 2020;140. doi:10.1016/j.ijmedinf.2020.104163

7. Ratnovsky A, Rozenes S, Bloch E, Halpern P. Statistical learning methodologies and admission prediction in an emergency department. Australas Emerg Care. 2021;24: 241–247. doi:10.1016/j.auec.2020.11.004

8. Spangler D, Hermansson T, Smekal D, Blomberg H. A validation of machine learning-based risk scores in the prehospital setting. PLoS One. 2019;14: e0226518. doi:10.1371/journal.pone.0226518

9. Mohanty SD, Lekan D, McCoy TP, Jenkins M, Manda P. A multi-modal machine learning approach towards predicting patient readmission. Park T, Cho YR, Hu X, Yoo I, Woo HG, Wang J, et al., editors. 2020 IEEE INTERNATIONAL CONFERENCE ON BIOINFORMATICS AND BIOMEDICINE. Univ North Carolina Greensboro, Dept Comp Sci, Greensboro, NC 27412 USA; 2020. pp. 2027–2035. doi:10.1109/BIBM49941.2020.9313588

10. Fenn A, Davis C, Buckland DM, Kapadia N, Nichols M, Gao M, et al. Development and Validation of Machine Learning Models to Predict Admission From Emergency Department to Inpatient and Intensive Care Units. Ann Emerg Med. 2021;78: 290–302. doi:10.1016/j.annemergmed.2021.02.029

11. Zhao P, Yoo I, Naqvi SH. Early Prediction of Unplanned 30-Day Hospital Readmission: Model Development and Retrospective Data Analysis. JMIR Med Inf 2021;9(3)e16306 https//medinform.jmir.org/2021/3/e16306. 2021;9: e16306. doi:10.2196/16306

12. Jayousi, Rashid; Assaf R. 30-day Hospital Readmission Prediction using MIMIC Data. The Institute of Electrical and Electronics Engineers, Inc. (IEEE) Conference Proceedings. Al-Quds University,Jerusalem,Palestine: The Institute of Electrical and Electronics Engineers, Inc. (IEEE); 2020. pp. 1–6. doi:http://dx.doi.org/10.1109/AICT50176.2020.9368625

13. Zebin T, Chaussalet TJ. Design and implementation of a deep recurrent model for prediction of readmission in urgent care using electronic health records. The Institute of Electrical and Electronics Engineers, Inc. (IEEE) Conference Proceedings. Piscataway: The Institute of Electrical and Electronics Engineers, Inc. (IEEE); 2019. pp. 1–5. Available: https://www.proquest.com/conference-papers-proceedings/design-implementation-deep-recurrent-model/docview/2270191328/se-2?accountid=17260

14. Curto S, Carvalho JP, Salgado C, Vieira SM, Sousa JMC. Predicting ICU readmissions based on bedside medical text notes. The Institute of Electrical and Electronics Engineers, Inc. (IEEE) Conference Proceedings. Piscataway: The Institute of Electrical and Electronics Engineers, Inc. (IEEE); 2016. pp. 2144-a-2151-h. Available: https://www.proquest.com/conference-papers-proceedings/predicting-icu-readmissions-based-on-bedside/docview/1839181633/se-2?accountid=17260

15. Feretzakis G, Karlis G, Loupelis E, Kalles D, Chatzikyriakou R, Trakas N, et al. Using Machine Learning Techniques to Predict Hospital Admission at the Emergency Department. J Crit Care Med. 2022;8: 107–116. doi:10.2478/jccm-2022-0003

16. Futoma J, Morris J, Lucas J. A comparison of models for predicting early hospital readmissions. J Biomed Inform. 2015;56: 229–238. doi:10.1016/j.jbi.2015.05.016

17. Holloway J, Neely C, Yuan X, Zhang Y, Ouyang J, Cantrell D, et al. Evaluating the performance of a predictive modeling approach to identifying members at high-risk of hospitalization. J Med Econ. 2020;23: 228–234. doi:10.1080/13696998.2019.1666854

18. Ko M, Chen E, Agrawal A, Rajpurkar P, Avati A, Ng A, et al. Improving hospital readmission prediction using individualized utility analysis. J Biomed Inform. 2021;119: 103826. doi:10.1016/j.jbi.2021.103826

19. Schiltz NK, Dolansky MA, Warner DF, Stange KC, Gravenstein S, Koroukian SM, et al. Impact of Instrumental Activities of Daily Living Limitations on Hospital Readmission: an Observational Study Using Machine Learning. J Gen Intern Med. 2020;35: 2865–2872. doi:http://dx.doi.org/10.1007/s11606-020-05982-0

20. van Walraven C, Dhalla IA, Bell C, Etchells E, Stiell IG, Zarnke K, et al. Derivation and validation of an index to predict early death or unplanned readmission after discharge from hospital to the community. CMAJ. 2010;182: 551–7. doi:10.1503/cmaj.091117

21. Billings J, Blunt I, Steventon A, Georghiou T, Lewis G, Bardsley M. Development of a predictive model to identify inpatients at risk of re-admission within 30 days of discharge (PARR-30). BMJ Open. 2012;2. doi:10.1136/bmjopen-2012-001667

22. Shulan M, Gao K, Moore CD. Predicting 30-day all-cause hospital readmissions. Health Care Manag Sci. 2013;16: 167–175. doi:10.1007/s10729-013-9220-8

23. Knaus WA, Draper EA, Wagner DP, Zimmerman JE. APACHE II: a severity of disease classification system. Crit Care Med. 1985;13: 818–829. Available: http://europepmc.org/abstract/MED/3928249

24. Knaus WA, Wagner DP, Draper EA, Zimmerman JE, Bergner M, Bastos PG, et al. The APACHE III prognostic system: Risk prediction of hospital mortality for critically III hospitalized adults. Chest. 1991;100: 1619–1636. doi:http://dx.doi.org/10.1378/chest.100.6.1619

25. González J, Soltero R. Emergency Severity Index (ESI) triage algorithm: trends after implementation in the emergency department. Bol Asoc Med P R. 2009;101: 7–10. Available: http://www.ncbi.nlm.nih.gov/pubmed/20120978

26. Physicians RC of. National Early Warning Score (NEWS) - Standardising the Assessment of Acute-Illness Severity in the NHS. London, England; 2012. Available: eISBN 978-1-86016-472-9

27. Dialog H, Wennberg D, Siegel M, Darin B, Filipova N, Russell R, et al. COMBINED PREDICTIVE MODEL Final report. Cambridge; 2006. Available: https://www.kingsfund.org.uk/sites/default/files/field/field_document/PARR-combined-predictive-model-final-report-dec06.pdf

28. Dinh MM, Russell SB, Bein KJ, Rogers K, Muscatello D, Paoloni R, et al. The Sydney Triage to Admission Risk Tool (START) to predict Emergency Department Disposition: A derivation and internal validation study using retrospective state-wide data from New South Wales, Australia. BMC Emerg Med. 2016;16: 46. doi:10.1186/s12873-016-0111-4

29. Charlson ME, Pompei P, Ales KL, MacKenzie CR. A new method of classifying prognostic comorbidity in longitudinal studies: Development and validation. J Chronic Dis. 1987;40: 373–383. doi:10.1016/0021-9681(87)90171-8

30. Donze J, Schnipper JL, Aujesky D. Potentially avoidable 30-day hospital readmissions in medicine patients: Derivation and validation of a prediction model. J Gen Intern Med. 2012;27: S275–S276. Available: http://ovidsp.ovid.com/ovidweb.cgi?T=JS&PAGE=reference&D=emed13&NEWS=N&AN=71296850

31. El Morr C, Ginsburg L, Nam S, Woollard S. Assessing the Performance of a Modified LACE Index (LACE-rt) to Predict Unplanned Readmission After Discharge in a Community Teaching Hospital. Interact J Med Res. 2017;6: e2. doi:10.2196/ijmr.7183

32. Le Gall JR. A new Simplified Acute Physiology Score (SAPS II) based on a European/North American multicenter study. JAMA J Am Med Assoc. 1993;270: 2957–2963. doi:10.1001/jama.270.24.2957

33. Ferrier D, Diver F, Corin S, McNair P, Cheng C. HealthLinks: Incentivising better value chronic care in Victoria. Int J Integr Care. 2017;17: 129. doi:10.5334/ijic.3241

34. Baig MM, Hua N, Zhang E, Robinson R, Spyker A, Armstrong D, et al. A machine learning model for predicting risk of hospital readmission within 30 days of discharge: validated with LACE index and patient at risk of hospital readmission (PARR) model. Med Biol Eng Comput. 2020;58: 1459–1466. doi:10.1007/s11517-020-02165-1

35. Lin C, Hsu S, Lu H-F, Pan L-F, Yan Y-H. Comparison of Back-Propagation Neural Network, LACE Index and HOSPITAL Score in Predicting All-Cause Risk of 30-Day Readmission. Risk Manag Healthc Policy. 2021;14: 3853–3864. doi:10.2147/RMHP.S318806

36. Jamei M, Nisnevich A, Wetchler E, Sudat S, Liu E. Predicting all-cause risk of 30-day hospital readmission using artificial neural networks. PLoS One. 2017;12: e0181173. doi:10.1371/journal.pone.0181173

37. Yu K, Xie X. Predicting Hospital Readmission: A Joint Ensemble-Learning Model. IEEE J Biomed Heal Informatics. 2020;24: 447–456. doi:10.1109/JBHI.2019.2938995

38. Tse Lin Ho E, En-Howe Tan I, Lee I, Yubai Wu P, Chong HF. Predicting Readmission at Early Hospitalization Using Electronic Health Data: A Customized Model Development. International Journal of Integrated Care (IJIC). SingHealth Regional Health System, Singapore: Ubiquity Press; 2017. pp. 1–2. doi:10.5334/ijic.3826

39. Yu S, Farooq F, van Esbroeck A, Fung G, Anand V, Krishnapuram B. Predicting readmission risk with institution-specific prediction models. Artif Intell Med. 2015;65: 89–96. doi:10.1016/j.artmed.2015.08.005

40. Wee Lai K, Wai Yong C, Pingguan-Murphy B, Huang Chuah J, Tee CATH. Prediction of Hospital Readmission Combining Rule-based and Machine Learning Model. The Institute of Electrical and Electronics Engineers, Inc. (IEEE) Conference Proceedings. University of Malaya,Faculty of Engineering,Dept. of Biomedical Engineering,Kuala Lumpur,Malaysia ; University of Malaya,Faculty of Engineering,Dept. of Electrical Engineering,Kuala Lumpur,Malaysia: The Institute of Electrical and Electronics Engineers, Inc. (IEEE); 2020. pp. 352–355. doi:http://dx.doi.org/10.1109/ICS51289.2020.00076

41. Tong L, Erdmann C, Daldalian M, Li J, Esposito T. Comparison of predictive modeling approaches for 30-day all-cause non-elective readmission risk. BMC Med Res Methodol. 2016;16: 26. doi:10.1186/s12874-016-0128-0

42. Mesgarpour M, Chaussalet T, Chahed S. Ensemble Risk Model of Emergency Admissions (ERMER). Int J Med Inform. 2017;103: 65–77. doi:10.1016/j.ijmedinf.2017.04.010

43. Rendell K, Koprinska I, Kyme A, Ebker‐White AA, Dinh MM. The Sydney Triage to Admission Risk Tool (START2) using machine learning techniques to support disposition decision‐making. Emerg Med Australas. 2019;31: 429–435. doi:10.1111/1742-6723.13199

44. Levin S, Toerper M, Hamrock E, Hinson JS, Barnes S, Gardner H, et al. Machine-Learning-Based Electronic Triage More Accurately Differentiates Patients With Respect to Clinical Outcomes Compared With the Emergency Severity Index. Ann Emerg Med. 2018;71: 565-574.e2. doi:10.1016/J.ANNEMERGMED.2017.08.005

45. Fialho AS, Cismondi F, Vieira SM, Reti SR, Sousa JMC, Finkelstein SN. Data mining using clinical physiology at discharge to predict ICU readmissions. Expert Syst Appl. 2012;39: 13158–13165. doi:10.1016/J.ESWA.2012.05.086

46. Mesgarpour M, Chaussalet T, Chahed S. Ensemble Risk Model of Emergency Admissions (ERMER). Int J Med Inform. 2017;103: 65–77. doi:10.1016/J.IJMEDINF.2017.04.010

47. Sushmita S, Khulbe G, Hasan A, Newman S, Ravindra P, Roy SB, et al. Predicting 30-Day Risk and Cost of “All-Cause” Hospital Readmissions. AAAI Work - Tech Rep. 2016; 453–461. Available: http://www.cms.gov/Medicare/Medicare-Fee-for-Service-

48. Chandra A, Rahman PA, Sneve A, McCoy RG, Thorsteinsdottir B, Chaudhry R, et al. Risk of 30-Day Hospital Readmission Among Patients Discharged to Skilled Nursing Facilities: Development and Validation of a Risk-Prediction Model. J Am Med Dir Assoc. 2019;20: 444-450.e2. doi:10.1016/j.jamda.2019.01.137

49. Hegselmann S, Ertmer C, Volkert T, Gottschalk A, Dugas M, Varghese J. Development and validation of an interpretable 3 day intensive care unit readmission prediction model using explainable boosting machines. Front Med. 2022;9. doi:10.3389/fmed.2022.960296

50. Conilione P, Jessup R, Gust A. Novel machine learning model for predicting multiple unplanned hospitalisations. BMJ Heal Care Inf. 2023;30: e100682. doi:10.1136/bmjhci-2022-100682

51. Azad TD, Ehresman J, Ahmed AK, Staartjes VE, Lubelski D, Stienen MN, et al. Fostering reproducibility and generalizability in machine learning for clinical prediction modeling in spine surgery. Spine J. 2021;21: 1610–1616. doi:10.1016/j.spinee.2020.10.006

52. Yang J, Soltan AAS, Clifton DA. Machine learning generalizability across healthcare settings: insights from multi-site COVID-19 screening. npj Digit Med. 2022;5: 69. doi:10.1038/s41746-022-00614-9

53. Murad MH, Katabi A, Benkhadra R, Montori VM. External validity, generalisability, applicability and directness: a brief primer. BMJ Evidence-Based Med. 2018;23: 17–19. doi:10.1136/ebmed-2017-110800

54. Wiens J, Shenoy ES. Machine Learning for Healthcare: On the Verge of a Major Shift in Healthcare Epidemiology. Clin Infect Dis. 2018;66: 149–153. doi:10.1093/cid/cix731

55. Ryu B, Yoo S, Kim S, Choi J. Development of Prediction Models for Unplanned Hospital Readmission within 30 Days Based on Common Data Model: A Feasibility Study. Methods Inf Med. 2021. doi:10.1055/s-0041-1735166

56. Ta WA, Goh HL, Tan CS, Sun Y, Aung KCY, Teoh ZW, et al. Development and implementation of nationwide predictive model for admission prevention: System architecture & machine learning. The Institute of Electrical and Electronics Engineers, Inc. (IEEE) Conference Proceedings. Piscataway: The Institute of Electrical and Electronics Engineers, Inc. (IEEE); 2018. pp. 303–306. Available: https://www.proquest.com/conference-papers-proceedings/development-implementation-nationwide-predictive/docview/2023820356/se-2?accountid=17260

57. Ta AWA, Goh HL, Ang C, Koh LY, Poon K, Miller SM. Two Singapore public healthcare AI applications for national screening programs and other examples. Heal Care Sci. 2022;1: 41–57. doi:10.1002/hcs2.10

58. Cabitza F, Campagner A, Soares F, García de Guadiana-Romualdo L, Challa F, Sulejmani A, et al. The importance of being external. methodological insights for the external validation of machine learning models in medicine. Comput Methods Programs Biomed. 2021;208: 106288. doi:10.1016/j.cmpb.2021.106288

59. Bousquet N. Diagnostics of prior-data agreement in applied Bayesian analysis. J Appl Stat. 2008;35: 1011–1029. doi:10.1080/02664760802192981

60. Rieke N, Hancox J, Li W, Milletarì F, Roth HR, Albarqouni S, et al. The future of digital health with federated learning. npj Digit Med. 2020;3: 119. doi:10.1038/s41746-020-00323-1

61. Antunes RS, André da Costa C, Küderle A, Yari IA, Eskofier B. Federated Learning for Healthcare: Systematic Review and Architecture Proposal. ACM Trans Intell Syst Technol. 2022;13: 1–23. doi:10.1145/3501813

62. Yoo JH, Jeong H, Lee J, Chung T-M. Open problems in medical federated learning. Int J Web Inf Syst. 2022;18: 77–99. doi:10.1108/IJWIS-04-2022-0080

63. Sheller MJ, Edwards B, Reina GA, Martin J, Pati S, Kotrotsou A, et al. Federated learning in medicine: facilitating multi-institutional collaborations without sharing patient data. Sci Rep. 2020;10: 12598. doi:10.1038/s41598-020-69250-1

64. Zhang A, Xing L, Zou J, Wu JC. Shifting machine learning for healthcare from development to deployment and from models to data. Nat Biomed Eng. 2022;6: 1330–1345. doi:10.1038/s41551-022-00898-y
