# Supplementary material for: Using machine learning methods to predict all-cause somatic hospitalizations in adults: A systematic review
Source: PLoS One. 2024 Aug 23;19(8):e0309175. doi: 10.1371/journal.pone.0309175 (PMC11343463; doi:10.1371/journal.pone.0309175)
Supplement: Attachment — Submitted filename: Response to reviewers.docx [file pone.0309175.s003.docx]

*Mohsen Askar*

*Hansine Hansens veg 18*

*Tromsø, Troms, 9019*

***Response to reviewers***

Manuscript title:” Using machine learning methods to predict all-cause somatic hospitalizations in adults: A systematic review.”

PONE-D-24-04075

Dear PLOS ONE Editor(s),

Thanks for the considering our work for publishing and for providing feedback on the manuscript. We appreciate the thoughtful comments and have addressed each of the suggestions to improve the manuscript content. We have responded to all comments, both related to the journal requirements and reviewers’ revision. Below are these comments, followed by our reply in red.

The lines’ numbers are highlighted in yellow. Lines’ numbers in the *original text* refer to their place in the previous submission, while lines’ numbers in the *revised text* refer to their place in the 'Revised Manuscript with Track Changes'.

**A) Journal Requirements:**

1. When submitting your revision, we need you to address these additional requirements.

Please ensure that your manuscript meets PLOS ONE's style requirements, including those for **file naming**. The PLOS ONE style templates can be found at 
<https://journals.plos.org/plosone/s/file?id=wjVg/PLOSOne_formatting_sample_main_body.pdf> and 
<https://journals.plos.org/plosone/s/file?id=ba62/PLOSOne_formatting_sample_title_authors_affiliations.pdf>

**Answer:**

The mentioned templates were checked and the title page, manuscript text, file naming was modified accordingly. Figures were also checked using PACE tool and converted to the accepted formats.

2. Thank you for stating the following financial disclosure: 
The publication charges for this article have been funded by a grant from the publication fund of UiT The Arctic University of Norway.
Please state what role the funders took in the study.  If the funders had no role, please state: "The funders had no role in study design, data collection and analysis, decision to publish, or preparation of the manuscript." 
If this statement is not correct you must amend it as needed. 
Please include this amended Role of Funder statement in your cover letter; we will change the online submission form on your behalf.

**Answer:**

Thank you for the clarification. The grant from UiT is just to pay the publication fee. The UiT has not been involved with any role in conducting the study. The funding statement was thus modified as recommended.

3. When completing the data availability statement of the submission form, you indicated that you will make your data available on acceptance. We strongly recommend all authors decide on a data sharing plan before acceptance, as the process can be lengthy and hold up publication timelines. Please note that, though access restrictions are acceptable now, your entire data will need to be made freely accessible if your manuscript is accepted for publication. This policy applies to all data except where public deposition would breach compliance with the protocol approved by your research ethics board. If you are unable to adhere to our open data policy, please kindly revise your statement to explain your reasoning and we will seek the editor's input on an exemption. Please be assured that, once you have provided your new statement, the assessment of your exemption will not hold up the peer review process.

**Answer:**

Thanks for comment. To comply with PLOS ONE’s open data policy, we provided the data extracted from the studies (CHARMS), the features used in the studies’ prediction models, TRIPOD, and PROBAST checklists assessments in the supplementary information (S2, Excel file) along with the proper citations to the all the studies included in the review. All statistics and calculations mentioned in the results section can be derived from the mentioned supplementary file. We changed the response of data availability statement to be “available” referring to the supporting information of the article.

**B) Reviewers' comments:**

**Reviewer #1**:

**Comments on the Introduction section (comments 1-3)**

As these three comments are related to the Introduction section, we responded to the three comments and provided the revised text of this part in the introduction after comment 3.

**1.** While the introduction mentions the shift towards ML techniques, providing a brief rationale for why ML is superior to traditional regression models and specific risk indexes would strengthen the argument. For example, it could be helpful to highlight ML’s capacity to manage complex, high-dimensional datasets and identify nonlinear relationships.

**Answer:**

Thanks for the comment. We agree with the reviewer that integrating the rationale of ML models' superiority over traditional regression and sole use of risk score in the introduction will strengthen the argument and enhance clarity. We also touched on that in the discussion section under “*Models’ performance comparisons*” lines 376-386 in the submitted manuscript. We revised the introduction text and added some references in the revised text (below comment 3).

**2.** The introduction cites studies that highlight the superiority of ML techniques. Providing a brief summary or key findings from these reviews would place the current study in the perspective of the existing literature and help readers understand the progression of research in the field of AI/ML.

**Answer:**

We agree with the reviewer, in the revised version we offered some a summary for key findings from the mentioned reviews to allow the reader to better understand the key findings of the prior reviews (see the revised text below comment 3).

**3.** In lines 53-54, authors have mentioned that recent reviews have proposed a framework for ML model development. This point needs to elaborated further.

**Answer:**

We briefly summarized the framework proposed in the mentioned study. (see the revised version of the introduction below).

**The original text** “Recent reviews concluded that ML techniques can improve readmission prediction ability over traditional statistical models [10,11], highlighted the importance of demonstrating the clinical usefulness of the models in practice, and proposed a framework for ML model development [12].” Lines 51-54

**The revised version of this part in the introduction** “Recent reviews emphasized the growing importance and effectiveness of ML models in predicting clinical outcomes such as hospital readmissions. These reviews concluded that ML techniques can improve readmission prediction ability over traditional statistical models. This improvement could be explained by ML models offering several advantages over traditional regression models such as flexibility, the ability to handle large, complex, high dimensional datasets, and identifying non-linear relationships [10]. These advantages can lead to improved predictive performance and better adaptability to diverse data sources. The reviews also highlighted the critical role of selecting features and addressed some challenges such as transparency, the difficulty of ML models’ interpretation, and the importance of handling class imbalance to enhance the models’ performance. Moreover, they highlighted the importance of demonstrating the clinical usefulness of the models in practice [11–13]. A systematic analysis of readmission prediction literature proposed a comprehensive framework for ML model development detailing steps from data preparation and preprocessing to suggesting methods of feature selection and transformation, data splitting, model training, validation, and evaluation [14]”. Lines 51-65

**Comments on the Methods section (comments 4-5)**
**4.** In the methods section, while the authors have mentioned the removal of duplicate studies, providing details on the methodology used for duplicate removal (e.g., software/tool used) would enhance transparency of methods.

**Answer:**

We agree with the reviewer, we used Mendeley reference manager to remove the duplicated studies. In cases where the reference manager showed uncertainty (very few studies), we manually checked and removed the duplicates. We edited the text in the methods section accordingly.

**The original text** “Duplicate studies were removed.” Line 106

**The revised text** “Duplicate studies were removed using Mendeley reference manager. In cases where the reference manager was uncertain, we manually checked and removed any duplicates”. Lines 121,122

**5.** The authors should consider providing a brief explanation of the PROBAST tool and its domains to help readers understand how bias and applicability were assessed.

**Answer:**

Thank you for the comment. We edited the text to include a brief explanation of what the PROBAST tool is and what domains it covers.

**The original text** “Despite that the main purpose of the review is descriptive, we assessed the risk of bias and applicability using the Prediction model Risk of Bias Assessment Tool (PROBAST) [22] by MA and MT. Four domains of risk of bias were evaluated. If any domain was not rated “low”, the overall risk of bias was considered “high”. Abstracts were not assessed due to their limited information.” Lines 119-123

**The revised text** “Despite that the main purpose of the review is descriptive, we assessed the risk of bias and applicability using the Prediction model Risk of Bias Assessment Tool (PROBAST) [22] by MA and MT. PROBAST is a commonly used tool to assess prediction models. The tool evaluates four domains; namely: Participants, Predictors, Outcome, and Analysis. For each domain, there is a set of questions to help judge the risk of both bias and applicability concerns. If any domain was not rated “low”, the overall risk of bias was considered “high”. Abstracts were not assessed due to their limited information. The assessment is attached to supplementary 2, sheet: PROBAST.” Lines 141-147

**Comments on the Results section (comment 6)**

**6.** Line 162: In population characteristics, the authors can include total number of patients, and other baseline characteristics from the included studies.

**Answer:**

Thanks for the comment. We included the individual population characteristics (e.g. number of unique patients, records (if mentioned), and age range) for the review studies in supplementary 2: sheet CHARMS. However, we believe that including the total number of patients of all studies would not be accurate for some reasons. For example, counting the patient population of all studies will likely result in repetition for example for the studies used MIMIC dataset “II, III” population either in total or partially and it would be difficult to distinguish the repeated from the unrepeated patients. Similarly, some studies used register data (either claim or health registers) which could include the whole population of a specific country while other registers (performed in the same country) has included patients from specific hospital or region, making it difficult to identify unique patients.

In the results section, we mentioned some useful statistics there such as reporting the unique patients, longitudinal records, the studies population sample size range, and the reported age. This is to provide the reader with a general understanding of the variability in the reporting quality and study size. For more detailed information for the individual studies, readers can refer to the mentioned supplementary material.

**Comments on the Discussion section (comment 7-13)**

**7.** Line 306-310: This can be highlighted as a limitation of this study, and can be elaborated further. Moreover, future directions are required as to how future studies can particularly tackle this.

**Answer:**

We agree that this could limit the study results. We edited the limitation part of the discussion section accordingly.

**The original text** “We identified the relevant literature from eight databases, but we have not approached authors for missing information on the studies. This is due to the considerable amount of missing information which could have impacted the assessment of bias risk. Assessing the quality of studies was also limited by not being able to access their codes.” Lines 438-441

**The revised text** “We identified the relevant literature from eight databases, but we have not approached authors for missing information on the studies. This is due to the considerable amount of missing information which could have impacted the assessment of bias risk. Our results are also limited by the fact that most of the reviewed studies were based on data from the USA which limits the generalizability because of the differences between populations and healthcare systems between countries. To address this limitation, future studies should aim to include diverse datasets from various countries and healthcare settings. Additionally, more efforts should be directed to compare models from different populations and settings to understand their limitations in different contexts. Assessing the quality of studies was also limited by not being able to access their code scripts.” Lines 540-549

**8.** Line 328, the sentence lacks a necessary comma. To improve clarity, it should be as follows: "The most used feature groups were demographics, diagnoses, physiological measurements, and laboratory tests, respectively."

**Answer:**

Yes, that is correct. We have fixed that.

**9.** In the "datasets and features" section of the discussion, the authors made a noteworthy observation regarding the increasing use of Natural Language Processing (NLP) techniques in recent studies. This trend shows a promising avenue with regard to utilizing textual data in predicting hospitalizations. Therefore, it is suggested to further discuss the potential benefits and challenges associated with employing NLP techniques for extracting information from clinical text.

**Answer:**

Thanks for the suggestions. We truly agree that NLP techniques are a very promising research direction, especially with the recent advancements in foundational models and Large Language Models. We expanded this part of the discussion to include a brief description of some benefits and challenges related to implementing NLP techniques, summarizing the key findings of two relevant literature reviews, as follows:

**The original text** “We noticed an increase in applying NLP techniques in recent studies suggesting that utilizing textual data is a promising future direction for predicting hospitalizations.” Lines 339-341

**The revised text** “We noticed an increase in applying NLP techniques in recent studies suggesting that utilizing textual data is a promising future direction for predicting hospitalizations. Incorporating NLP techniques in prediction models will provide models with a rich source of clinical information that could not be present in the tabular format of patient records. It will also improve the research scalability by the automatic extraction of relevant information rather than manual processing. Furthermore, it can provide real-time assistance for clinicians. However, some challenges should be considered such as the limited availability of sharing large, annotated datasets which are necessary for developing efficient NLP models, the current popular evaluation methods may not be clinically relevant, and the lack of transparent protocols to ensure NLP methods are reproducible [63,64].” Lines 398-407

**10.** In lines 340-351, the authors noted that a few studies reported handling class imbalance in the dataset and that resampling techniques, especially under sampling were the most used approach. To enhance reader comprehension, it would be beneficial to succinctly define and explain these techniques for those who may not be familiar with the terminology, thus improving overall reader understanding.

**Answer:**

We appreciate the reviewer’s suggestion to enhance reader comprehension. In the revised version of text below, we added a short definition of class imbalance, why is it a problem and a short description of handling class imbalance techniques along with some relevant references.

**The original text** “Only a few studies (32 studies) reported handling class imbalance in the dataset. Resampling techniques, especially undersampling were the most used approach. It should be noted that resampling techniques have some drawbacks such as overfitting or losing useful information which can introduce problematic consequences and hinder model learning [65,66].” Lines 349-353

**The revised text** “Only a few studies (32 studies) reported handling class imbalance in the dataset. Class imbalance means that the outcome contains more samples from one class (majority class) over the other classes (minority class) [65] and represents one of the most common issues in training ML models in predicting hospitalizations. However, it is not usually taken into consideration in the readmission risk prediction literature [66]. The problem with class imbalance is that the models could be biased towards the majority class leading to a misleadingly high prediction performance [67]. Resampling techniques, especially undersampling were the most used approach. Resampling techniques involve balancing the distribution of outcome classes either by oversampling or undersampling. Oversampling involves increasing the number of instances of minority class (e.g. SMOTE), while undersampling involves randomly reducing the number of majority class instances, thus balancing the class distribution [68]. It should be noted that resampling techniques have some drawbacks such as overfitting or losing useful information which can introduce problematic consequences and hinder model learning [69,70].” Lines 415-428

**11.** Similarly, in section ‘Models’ performance comparisons’, the authors mentioned a key finding that tree-based boosting algorithms often outperformed other algorithms. I suggest that the authors briefly explain what these algorithms are, how they work, significance of these algorithms and why they outperformed others.

**Answer:**

Thanks for the reviewer for the suggestion. In the revised version of the text, we briefly explain what the boosting family algorithms are, how to work, and possible reasons why they could perform better.

**The original text** “…, we found that tree-based boosting algorithms often outperformed other algorithms (Table 2, Fig 5)” Lines 362-363

**The revised text** “…, we found that tree-based boosting algorithms often outperformed other algorithms (Table 2, Fig 5). Tree-based boosting algorithms such as Gradient Boosting Machine (GBM), XGBoost, and AdaBoost, are a class of ensemble learning methods that build multiple decision trees sequentially [73]. Each new decision tree corrects the errors of previous ones by giving more focus to samples that were difficult to estimate [74]. The predictions of the trees are then combined to produce the final model prediction [75]. This group of algorithms has many advantages such as training multiple models which enhances the prediction performance over training a single one, flexibility to handle different data types, capturing non-linear patterns, and being less prone to overfitting [76].” Lines 439-447

**12.** In lines 399-400, the authors state that” In Supplementary 1: S6, we summarize the problems facing EV and the generalization of ML models along with some suggested solutions.” It would enhance the significance of the discussion to incorporate a brief overview of these challenges and solutions.

**Answer:**

We revised the text to provide a brief and concise summary of S1: 6 in the supplementary material.

**The original text** “… In Supplementary 1: S6, we summarize the problems facing EV and the generalization of ML models along with some suggested solutions.” Lines 399-400

**The revised text** “However, there are still several obstacles facing ML models’ generalizability. These obstacles can be categorized as either model-related or data-related. Model-related obstacles include issues with transparency in model development and results reporting. Data-related ones include the diversity of data structure, formats, population, etc. across different healthcare systems, and the lack of a standardized data preprocessing framework. Additionally, the strict health data privacy regulations.

Adopting Common Data Models (CDMs) [94], designing a comprehensive and widely accepted framework for data preprocessing, and implementing Federated Learning (FL) [95] could help address these issues. In S1: 6, we provide a more detailed explanation of these obstacles and solutions.” Lines 485-494

**13.** In lines 417-418, the authors emphasize the pivotal role of open-source datasets like MIMIC-III [94] in boosting machine learning (ML) research within the healthcare domain. To underscore the significance of these datasets further, the authors could cite evidence or statistics demonstrating the widespread impact of these open-source datasets. This will support the need of leveraging open data for advancing ML applications in healthcare.

**Answer:**

Thanks for the comment. We edited the text to add some statistics and references that show the impact of MIMIC-III as publicly available dataset on academic research.

**The original text** “There is also no doubt that open-source datasets such as MIMIC-III [94], have boosted ML research and opened many chances to develop ML in the health domain.” Lines 417-418

**The revised text** “There is also no doubt that publicly available datasets such as MIMIC-III [108], have boosted ML research and opened many chances to develop ML in the health domain. MIMIC-III has been cited more than 3,000 times to date. The dataset has enabled numerous studies that focus on developing predictive models and enhancing clinical decision support systems [109,110].” Lines 516-520
