# Supplementary material for: Using machine learning methods to predict all-cause somatic hospitalizations in adults: A systematic review
Source: PLoS One. 2024 Aug 23;19(8):e0309175. doi: 10.1371/journal.pone.0309175 (PMC11343463; doi:10.1371/journal.pone.0309175)
Supplement: Attachment — Submitted filename: Response to reviewers.docx [file pone.0309175.s004.docx]

*Mohsen Askar*

*Hansine Hansens veg 18*

*Tromsø, Troms, 9019*

***Response to reviewers***

Manuscript title:” Using machine learning methods to predict all-cause somatic hospitalizations in adults: A systematic review.”

PONE-D-24-04075R1

Dear PLOS ONE Editor(s),

Thanks for the revision comments. We have addressed the suggestions and responded to all comments. Below are these comments, followed by our reply in red. The lines’ numbers are highlighted in yellow. Lines’ numbers in the *original text* refer to their place in the previous submission, while lines’ numbers in the *revised text* refer to their place in the 'Revised Manuscript with Track Changes'.

A) **Journal Requirements:**

Please review your reference list to ensure that it is complete and correct. If you have cited papers that have been retracted, please include the rationale for doing so in the manuscript text, or remove these references and replace them with relevant current references. Any changes to the reference list should be mentioned in the rebuttal letter that accompanies your revised manuscript. If you need to cite a retracted article, indicate the article’s retracted status in the References list and also include a citation and full reference for the retraction notice.

**Answer:**

The reference list was reviewed. We used the Retraction Watch Database (owned by Crossref) to check if any cited articles were retracted. To ensure the completeness of the citations and enhance the accuracy, we updated some references with their DOI numbers as follows in the table.

| **Reference number [27]:** | |
| --- | --- |
| Original citation | Donze J, Schnipper JL, Aujesky D. Potentially avoidable 30-day hospital readmissions in medicine patients: Derivation and validation of a prediction model. J Gen Intern Med. 2012;27: S275–S276. Available: http://ovidsp.ovid.com/ovidweb.cgi?T=JS&PAGE=reference&D=emed13&NEWS=N&AN=71296850 |
| Updated citation | Donzé J, Aujesky D, Williams D, Schnipper JL. Potentially Avoidable 30-Day Hospital Readmissions in Medical Patients. JAMA Intern Med. 2013;173: 632. doi:10.1001/jamainternmed.2013.3023 |
| **Reference number [47]:** | |
| Original citation | Lorenzana A, Tyagi M, Wang QC, Chawla R, Nigam S. Using text notes from call center data to predict hospitalization. Value Heal. 2016;19: A87. Available: http://ovidsp.ovid.com/ovidweb.cgi?T=JS&PAGE=reference&D=emed17&NEWS=N&AN=72311155 |
| Updated citation | Lorenzana A, Tyagi M, Wang QC, Chawla R, Nigam S. Using text notes from call center data to predict hospitalization. Value Heal. 2016;19: A87. doi:10.1016/j.jval.2016.03.1801 |
| **Reference number [53]:** | |
| Original citation | Curto S, Carvalho JP, Salgado C, Vieira SM, Sousa JMC. Predicting ICU readmissions based on bedside medical text notes. The Institute of Electrical and Electronics Engineers, Inc. (IEEE) Conference Proceedings. Piscataway: The Institute of Electrical and Electronics Engineers, Inc. (IEEE); 2016. pp. 2144-a-2151-h. Available: https://www.proquest.com/conference-papers-proceedings/predicting-icu-readmissions-based-on-bedside/docview/1839181633/se-2?accountid=17260 |
| Updated citation | Curto S, Carvalho JP, Salgado C, Vieira SM, Sousa JMC. Predicting ICU readmissions based on bedside medical text notes. 2016 IEEE International Conference on Fuzzy Systems (FUZZ-IEEE). Piscataway: IEEE; 2016. pp. 2144-a-2151-h. doi:10.1109/FUZZ-IEEE.2016.7737956 |
| **Reference number [59]:** | |
| Original citation | Boggan JC, Schulteis RD, Simel DL, Lucas JE. Use of a natural language processing algorithm to predict readmissions at a veterans affairs hospital. J Gen Intern Med. 2019;34: S396–S397. doi:http://dx.doi.org/10.1007/11606.1525-1497 |
| Updated citation | Boggan JC, Schulteis RD, Simel DL, Lucas JE. Use of a natural language processing algorithm to predict readmissions at a veterans affairs hospital. J Gen Intern Med. 2019;34: S396–S397. doi:10.1007/s11606-019-05007-5 |
| **Reference number [61]:** | |
| Original citation | Li Z, Xing X, Lu B, Li Z. Early Prediction of 30-day ICU Re-admissions Using Natural Language Processing and Machine Learning. arXiv.org. Ithaca: Cornell University Library, arXiv.org; 2019. Available: https://www.proquest.com/working-papers/early-prediction-30-day-icu-re-admissions-using/docview/2302243179/se-2 |
| Updated citation | Li Z, Xing X, Lu B, Zhao Y, Li Z. Early Prediction of 30-Day ICU Re-admissions Using Natural Language Processing and Machine Learning. Biomed Stat Informatics. 2019;4: 22. doi:10.11648/j.bsi.20190403.11 |
| **Reference number [96]:** | |
| Original citation | Yang C, Rangarajan A, Ranka S. Global Model Interpretation via Recursive Partitioning. |
| Updated citation | Yang C, Rangarajan A, Ranka S. Global Model Interpretation via Recursive Partitioning. 2018 [cited 27 Dec 2022]. Available: http://arxiv.org/abs/1802.04253 |
| **Reference number [98]:** | |
| Original citation | Du M, Liu N, Hu X. Techniques for Interpretable Machine Learning. 2008. |
| Updated citation | Du M, Liu N, Hu X. Techniques for interpretable machine learning. Commun ACM. 2019;63: 68–77. doi:10.1145/3359786 |
| **Reference number [99]:** | |
| Original citation | Wu CX, Suresh E, Phng FWL, Tai KP, Pakdeethai J, D’souza JLA, et al. Effect of a Real-Time Risk Score on 30-day Readmission Reduction in Singapore. Appl Clin Inform. 2021;12: 372–382. doi:10.1055/S-0041-1726422/ID/OR200228RA-71 |
| Updated citation | Wu CX, Suresh E, Phng FWL, Tai KP, Pakdeethai J, D’Souza JLA, et al. Effect of a Real-Time Risk Score on 30-day Readmission Reduction in Singapore. Appl Clin Inform. 2021;12: 372–382. doi:10.1055/s-0041-1726422 |

B) **Reviewers' comments:**

Reviewer #2:

- The authors have submitted a revised study on using machine learning methods to predict all-cause somatic hospitalizations in adults.
- the authors have extensively revised and addressed all the comments provided in the first round of revision.
- this study can benefit with a shorter introduction, which will improve readability of this paper.

**Answer:**

Thanks for the feedback. We believe the information and the flow of ideas mentioned in the introduction are needed for the readers to understand the need for conducting the review. We also agree on the importance of a concise introduction to improving readability. Therefore, we shortened and removed some sentences in the introduction which will not impact the overall flow and understanding.

- authors should consider mentioning company and version name for the referencing software of Mendeley.

**Answer:**

Thanks for the suggestion. We updated the manuscript text as follows:

**Original text:**

“Duplicate studies were removed using Mendeley reference manager.” Line 138

**Revised text:**

“Duplicate studies were removed using Mendeley Reference Manager (version 1.19.8, Elsevier).” Line 136-137

- instead of S2 or S3, authors can write Supplementary Table 3 or Supplementary Figure 2 etc etc.

**Answer:**

Thanks for the suggestion. We referred to the supplementary as S1, S2, etc. according to PLOS ONE manuscript formatting guidelines. However, to enhance the readability, we updated referring to supplementary material from S1:1, S1:2 to S1: Section 1, S1: Section 2, and so on. This is also to avoid the confusion between section numbers with page numbers.

- in my opinion, full forms of abbreviations mentioned in the table should be provided in a footnote or within somewhere in the title of the table, and not in S2 sheet.

**Answer:**

We agree with the reviewer that including the abbreviations in the footnote of the table will enhance the readability and make it easier for the readers. We edited the footnote to include all the abbreviations mentioned in the Table 2 arranged alphabetically (lines 269-278).

- the authors should submit a document with double spaced text to improve readability for the reviewers as well.

**Answer:**

Thank you for the feedback. We will resubmit the document with double-spaced text to enhance the document's readability.
